# Lysosomal quality control of cell fate: a novel therapeutic target for human diseases

**DOI:** 10.1038/s41419-020-03032-5

**Published:** 2020-09-30

**Authors:** Sheng-yu Zhu, Ren-qi Yao, Yu-xuan Li, Peng-yue Zhao, Chao Ren, Xiao-hui Du, Yong-ming Yao

**Affiliations:** 1 Trauma Research Center, Fourth Medical Center and Medical Innovation Research Division of the Chinese PLA General Hospital, 100048 Beijing, People’s Republic of China; 2 Department of General Surgery, First Medical Center of the Chinese PLA General Hospital, 100853 Beijing, People’s Republic of China; 3 School of Medicine, Nankai University, 300071 Tianjin, People’s Republic of China; 4 Department of Burn Surgery, Changhai Hospital, Naval Medical University, 200433 Shanghai, People’s Republic of China

**Keywords:** Cell biology, Diseases

## Abstract

In eukaryotic cells, lysosomes are digestive centers where biological macromolecules are degraded by phagocytosis and autophagy, thereby maintaining cellular self-renewal capacity and energy supply. Lysosomes also serve as signaling hubs to monitor the intracellular levels of nutrients and energy by acting as platforms for the assembly of multiple signaling pathways, such as mammalian target of rapamycin complex 1 (mTORC1) and adenosine 5′-monophosphate (AMP)-activated protein kinase (AMPK). The structural integrity and functional balance of lysosomes are essential for cell function and viability. In fact, lysosomal damage not only disrupts intracellular clearance but also results in the leakage of multiple contents, which pose great threats to the cell by triggering cell death pathways, including apoptosis, necroptosis, pyroptosis, and ferroptosis. The collapse of lysosomal homeostasis is reportedly critical for the pathogenesis and development of various diseases, such as tumors, neurodegenerative diseases, cardiovascular diseases, and inflammatory diseases. Lysosomal quality control (LQC), comprising lysosomal repair, lysophagy, and lysosomal regeneration, is rapidly initiated in response to lysosomal damage to maintain lysosomal structural integrity and functional homeostasis. LQC may be a novel but pivotal target for disease treatment because of its indispensable role in maintaining intracellular homeostasis and cell fate.

## Facts

- The lysosome functions as the terminal degradation site and a cellular sensor and signaling hub.
- Lysosomal damage is involved in different pathways of cell death.
- Lysosomal dysfunction is related to various human diseases.
- LQC may be a potential therapeutic target for human diseases.

## Open questions

- What are the effects of other nutrients, in addition to amino acids and growth factors, on lysosomal signaling?
- Is there a specific anchored receptor involved in lysophagy?
- Are there other mechanisms of cell death mediated by lysosomal damage, in addition to the release of cathepsins and hydrolases?

## Introduction

The lysosome was first discovered by de Duve, who defined it as a monolayer vesicle rich in acidic hydrolases^1^. It was previously found to be an extremely important intracellular degradation site, serving as the terminal destination during phagocytosis and autophagy^2^. It has been demonstrated that lysosomes are capable of sensing and addressing cellular signals and playing essential roles in coping with stress stimuli^3^. For example, lysosomes are programmed to monitor the intracellular levels of nutrients and energy, thus engaging in nutrient sensing, metabolic regulation, and cellular homeostatic maintenance by providing an initial platform for the activation of mammalian target of rapamycin complex 1 (mTORC1) and adenosine 5′-monophosphate (AMP)-activated protein kinase (AMPK)^4^. Damage to lysosomes not only disrupts cellular clearance ability but also results in the leakage of multiple enzymes, triggering the activation of a series of cell death pathways^5^. Lysosomal damage is involved in various forms of cell death, such as apoptosis^6,7^, necroptosis^8,9^, pyroptosis^10,11^, and ferroptosis^12,13^, hinting that it is of great significance to the clarification of the potential mechanism of lysosomal damage and further exploration of efficient treatments based on lysosomal quality control (LQC). Indeed, the structural and functional abnormalities of lysosomes are critical for the pathogenesis and development of various diseases, including tumors^14–16^, neurodegenerative diseases^17–19^, inflammatory diseases^20–22^, and cardiovascular diseases^23–25^, making LQC a novel but pivotal target for treatments. Endolysosome damage consists of a series of specific cellular responses to lysosomal damage, including lysosomal repair, lysophagy, and lysosomal regeneration^26^, which have been identified as being crucial for maintaining intracellular homeostasis.

## The structure and function of lysosomes

### Lysosomal structure

Lysosomes are monolayer membrane organelles surrounded by a 7–10-nm-thick lipid membrane. The membrane vacuolar-type H^+^-ATPase (V-ATPase) is a distinct characteristic of lysosomes and can continuously pump H^+^ into lysosomes to maintain an acidic environment^27^. In addition to V-ATPase, other membrane proteins, such as lysosome-associated membrane proteins (LAMPs), ion channels, and multiple transporters, are the molecular bases for lysosomal function^28^. For example, LAMPs account for 80% of lysosomal membrane proteins, protecting the lysosomal membrane from acidic digestion through their highly glycosylated intracavitary parts^29^. Lysosomal transporters are needed for transporting the final products into the cytoplasm for further metabolic utilization^30,31^.

The pH value of the lysosomal lumen is maintained within a range from 3.5 to 5.5, which provides an optimal environment for the degradation of biological macromolecules^3^. To date, more than 60 kinds of acidic hydrolysis enzymes have been isolated from lysosomes. In addition to hydrolases, lysosomes can act as a store for various ions. Ca^2+^ is important for the function of lysosomes, affecting lysosome movement, membrane transport and repair, nutrient sensing, and organelle membrane contact^32^. Other ions, such as Na^+^, K^+^, Cl^−^, and Zn^2+^, are required for lysosomal responses, including transportation, the maintenance of membrane potential, and the activation of lysosomal enzymes^33^.

### Lysosomal function

**Fig. 1:**
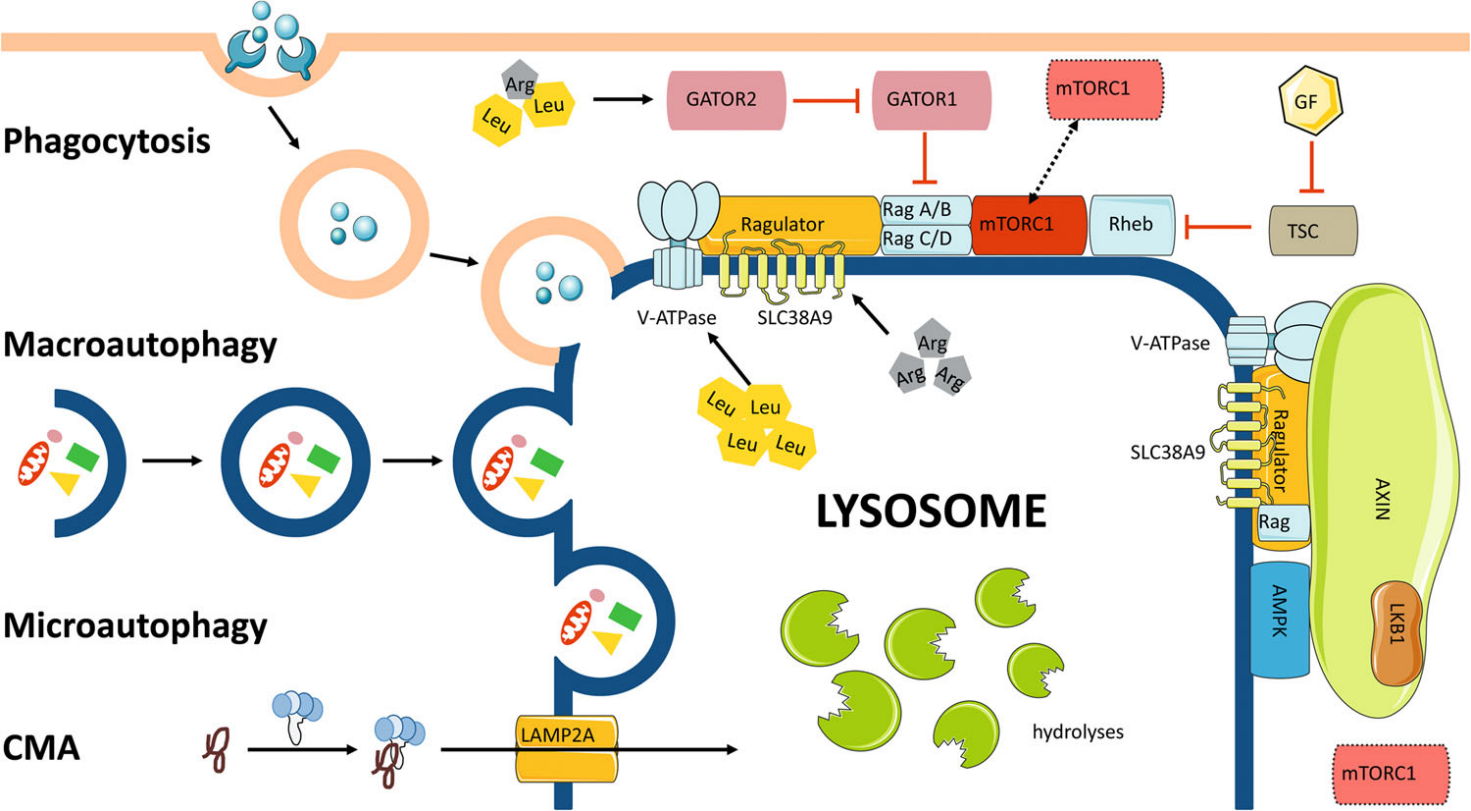
Lysosomal function. Lysosomes function as terminal sites of phagocytosis and autophagy and as cellular sensors and signaling hubs. Cargos are transported to lysosomes for degradation by the phagosome formed by the closure of reshaped plasma membrane during phagocytosis or the autophagosome formed by the closure of the phagophore in macroautophagy, directly swallowing into lysosomes through membrane indentation, protuberance, or the separation of the lysosomal membrane in microautophagy, and chaperone binding mainly through LAMP2A in CMA. Lysosomes also function as mTORC1 and AMPK signaling hubs. Under conditions of nutrient enrichment, Rag GTPases, heterodimers formed by RagA/B and RagC/D, are activated and tethered to the lysosomal membrane, further recruiting mTORC1. Rag GTPases can be activated by Ragulator and inhibited by the GATOR1 complex. Ragulator is activated by SLC38A and lysosomal V-ATPase, which stimulated by arginine and leucine in the lysosomal lumen, respectively, while the GATOR1 complex is inhibited by GATOR2, which is stimulated by amino acids in the cytoplasm. Then, mTORC1 is activated by Rheb GTPases stimulated by GF through the inhibition of TSC. Upon glucose deficiency, Axin causes the dissociation and inactivation of mTORC1 through the inhibition of Ragulator. In addition, Axin recruits AMPK by interacting with LKB1 and induces AMPK activation by forming complexes with V-ATPase and Ragulator.

Lysosomes are the main digestive centers of eukaryotes and can effectively degrade biological macromolecules to maintain self-renewal capacity and meet energy needs^2^. Extracellular materials, such as pathogens and toxins, can be transported to lysosomes for degradation through phagocytosis^34^, while intracellular cytoplasmic macromolecules, unfolded or misfolded proteins, and even whole organelles are captured and transported to lysosomes, mainly through autophagy^35,36^. Recent studies have indicated that lysosomes are the primary centers of metabolic signaling pathways, have critical involvement in nutrient sensing, and are capable of monitoring the cellular metabolic state and further facilitate adaption to nutrient deprivation by activating mTORC1 and AMPK^37–39^ (Fig. 1).

#### Phagocytosis and autophagy

Phagocytosis is programmed to eliminate invading pathogens and apoptotic cells^40^, making it crucial for maintaining intracellular homeostasis^41,42^. Cells undergo irreversible injury, and foreign stimuli, such as that from pathogens and associated toxins, are efficient in initiating phagocytosis^43^. Phagocytosis is completed by the following processes: the plasma membrane is reshaped by actin polymerization and local exocytosis, closing at the distal end to form early phagosomes^43^; then, to promote further digestion, phagosomes gradually fuse with lysosomes, which involves membrane remodeling, acidification of phagocytes, and the formation of oxidizing and degrading environments^44^.

Autophagy is characterized by vesicles swallowing cytoplasmic proteins or organelles and further fusing with lysosomes for content degradation, which is essential for intracellular metabolic homeostasis and the renewal of certain organelles^35^. Generally, autophagy is divided into macroautophagy, microautophagy, and chaperone-mediated autophagy (CMA)^45^. In contrast to macroautophagy, which is termed generalized autophagy, autophagosomes are not formed during microautophagy; instead, the lysosome directly swallows lysosomes through the indentation, protuberance or separation of the lysosomal membranes^46^. CMA is completed when a chaperone binds and transports a target protein to lysosomes for degradation^47^. Heat shock protein (Hsp) 70, the molecular chaperone that plays a critical role in CMA, is capable of recognizing and binding substrates for lysosomal degradation^48^. In addition, Hsp70 promotes the multimerization of LAMP type 2a, the lysosomal receptor involved in CMA, to form a translocation complex^49,50^, in which a translocation channel is further formed^51^ through which only unfolded substrates are permitted to pass^52^. Similarly, other molecular chaperones are required in CMA, including Hsp90, which regulates the stabilization of the translocation complex^49^, Hsp40, Hsp70-interacting protein (Hip), and Hsp70-Hsp90-organizing protein (Hop), which facilitates the translocation process^53^. Selective autophagy is programmed to degrade aggregates of proteins, invading pathogens, and damaged organelles, such as peroxisomes, mitochondria, lysosomes, nuclei, and endoplasmic reticula (ER)^54,55^. The ubiquitin-dependent sensor system is critical for targeting and degrading distinct substrates^56^. Autophagy-specific receptors further tether the ubiquitinated cargo to autophagosomes that carry microtubule-associated protein 1 light chain 3 (LC3)/GABAA receptor-associated protein (GABARAP) and integrate with lysosomes for degradation^57,58^.

#### Cellular sensor and signaling hub

Lysosomes are key hubs for integrating signals in response to nutrients and energy^37,39^. mTORC1 and AMPK, both of which are important metabolic regulators, assemble their signal centers on the surface of lysosomes to form a switch for anabolic and catabolic processes^37,39,59^.

mTORC1 is an important regulatory molecule of cell proliferation and metabolism because it senses intracellular signals, such as those from amino acids and growth factors, further triggering certain pathways to promote cell growth^60^. Intracellular signals can activate mTORC1 mainly through Rag GTPases and Ras homolog enriched in brain (Rheb) GTPases at the surface of lysosomes stimulated by amino acids and growth factors, respectively^61^. Rag GTPases are heterodimers formed by the combination of RagA/B and RagC/D, which can be tethered to the lysosomal membrane by Ragulator^61^. Under enrich nutrition conditions, the activation of Ragulator by amino acid signals enables Rag GTPases to bind and recruit mTORC1^61^. The changes in amino acids in the lysosomal lumen and cytoplasm are sensed by different mechanisms. In the lysosomal lumen, arginine and leucine facilitate the activation of Ragulator by stimulating SLC38A, a sodium-coupled amino acid transporter and lysosomal V-ATPase on the lysosomal membrane, thereby promoting the binding of Rag GTPases and mTORC1^62–64^. In the cytoplasm, amino acids, such as arginine and leucine, can lead to the activation of the GATOR2 complex, which further enhances the binding capacity of Rag GTPases and mTORC1 by inhibiting the hydrolysis of the GATOR1 complex by Rag GTPases^65,66^.

In the GTP-bound form, Rheb reportedly contributes to the activation of mTORC1 on the surface of lysosomes, and this process is disturbed by the activation of tuberous sclerosis complex (TSC)^67^. When the inhibitory status of TSC is induced by growth factors, the activation of mTORC1 augments cellular anabolism by stimulating the biosynthesis and inhibiting autophagy^60,68^. It was found that mTORC1 induces ribosomal biogenesis by phosphorylating and promoting the translation of S6 kinases^69^. The inhibitory effect of mTORC1 on autophagy is induced by phosphorylating and suppressing the nuclear translocation of transcription factor EB (TFEB)^70^.

AMPK is essential for the regulation of cellular metabolism. Upon glucose deficiency, Axin, a scaffolding protein that is capable of inhibiting the activity of Ragulator, triggers the dissociation and inactivation of mTORC1 on the lysosomal membrane^71^. In addition, Axin recruits AMPK by interacting with liver kinase B1 (LKB1) and induces AMPK activation by forming complexes with V-ATPase and Ragulator^4^. This activation of AMPK signaling reportedly enhances cellular energy storage processes by increasing glucose uptake, promoting autophagy, and inhibiting catabolism through the disruption to mTORC1 activation^72^.

## Lysosomal quality control

**Fig. 2:**
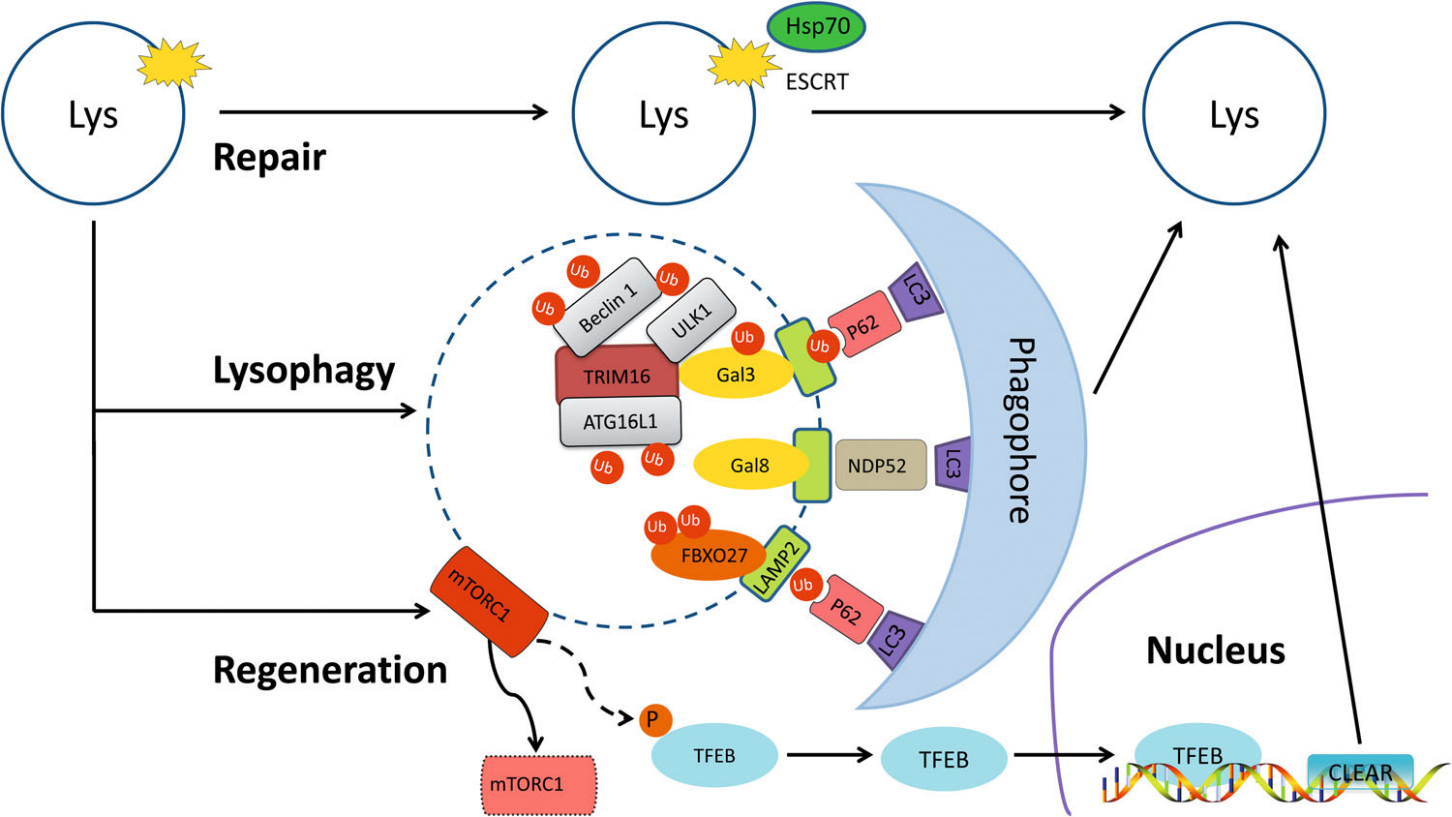
Lysosomal quality control. Lysosomal quality control includes lysosomal repair, lysophagy, and lysosomal regeneration. The damaged lysosomal membrane can be repaired by Hsp70 and ESCRT. In lysophagy, damaged lysosomes are ultimately transported to normal lysosomes for degradation through phagosomes formed by phagophores. Two pathways for damaged lysosomes have been identified: Gals and SCF^FBXO27^. After binding with TRIM16, Gal3 accumulates at damaged lysosomes and recruits ULK1, Beclin 1, and ATG16L1. Then, Gal3 is modified by the K63 ubiquitin chain and binds to LC3 on the phagophore via p62. Gal8 accumulates at damaged lysosomes and binds to LC3 on the phagophore via NDP52. SCF^FBXO27^ is recruited to damaged lysosomes and ubiquitinates LAMP2, thereby binding to LC3 on the phagophore via p62. In addition, the inhibition of mTORC1 by the loss of lysosomes can enable TFEB to bind with CLEAR and promote lysosomal generation.

Lysosomal membrane permeabilization (LMP) or lysosomal complete rupture is the common form of lysosomal damage, and it is a potential hazard to cell fate and function^73^. Maintaining lysosomal structural integrity and functional homeostasis through LQC favors the physiological functions of cells. Under normal conditions, glycosylation of lysosomal membrane proteins can effectively maintain membrane stability by preventing the lysosomal membrane from being destroyed by lumen proteolytic enzymes^28^. However, LMP with the loss of membrane integrity and increased permeability occur after persistent exposure to oxygen radicals, optical damage, and other factors^74,75^, thereby resulting in the release of cathepsins and hydrolases into the cytoplasm^76,77^. When the LMP is not repaired, persistent lysosomal rupture may lead to the massive release of lysosomal contents, cascade hydrolysis of cytoplasmic contents, and extensive cytoplasmic acidification, thus contributing to irreversible cell damage^76^. In response to various stimuli, endolysosome damage leads to the formation of specific cellular elements via efficient LQC, which includes lysosomal repair, lysophagy, and lysosomal regeneration^26^ (Fig. 2).

### Lysosomal repair

When LMP occurs, endogenous protective mechanisms are initiated to repair membrane damage, and prevent the release of lumenal hydrolases^73^. Previous studies documented the protective effects of Hsp70 for maintaining lysosomal integrity, showing that it can stabilize lysosomal membrane by binding to lipid bisphosphonates and enhancing the activity of acidic sphingolipase^78,79^. Recent studies showed that endosomal sorting complexes required for transport (ESCRT) was critical for the repair of damaged lysosomal membranes^80–82^. ESCRT is a highly conserved transport system commonly found in yeast and other eukaryotic cells, and it is programmed to transport ubiquitinated proteins into lysosomes^80^. ESCRTs can repair small perforations in the lysosomal membrane at an early stage of lysosomal damage, which are reportedly recruited in only a few minutes^80^. Further study revealed that the Ca^2+^ efflux from damaged lysosomes enhanced the lipid-binding activity of apoptosis-linked gene-2-interacting protein X (ALIX), a component of the ESCRT complex, for efficient binding to the lysosomal membrane. Moreover, Tsg101, another component of the ESCRT complex, was shown to be indispensable, as deficiency of ALIX or Tsg101 led to the inability to repair damaged lysosomes and irreversible cell death^81^. Currently, the precise mechanism by which ESCRT repairs lysosomal membranes is not clear, but it might be involved in inducing the formation of filamentous helices on the membrane surface and the contraction of pores in the lipid bilayer^82^.

### Lysophagy

When lysosomal membrane damage cannot be reversed, selective autophagy of lysosomes is initiated to ensure the effective clearance of damaged lysosomes, a process named lysophagy^73^. Similar to selective autophagy, the ubiquitination of impaired lysosomes is the major factor driving and modulating lysophagy^55^. Two ubiquitination pathways regulate lysophagy, and they involve galectins (Gals) and SCF^FBXO27^ ^26,83–85^. Gals are clearly crucial for lysophagy because they recognize glycoproteins in the lysosomal membrane after lysosomal rupture^26,83,84^. Gal3 is able to sense damaged lysosomes, where they accumulate to induce further lysophagy by recruiting autophagy regulatory factors, including uncoordinated-51-like kinase 1 (ULK1), Beclin 1, and autophagy-related protein 16L1 (ATG16L1) after binding with E3 ubiquitin ligase-tripartite domain containing protein 16 (TRIM16)^86^. Then, Gal3 is modified with a K63 ubiquitin chain and binds to LC3 on the phagophore through autophagy receptor p62, and the phagophore is encapsulated, forming an autophagosome for subsequent degradation^86^. Other galectins, such as Gal8 and Gal9, can also be recruited to damaged lysosomes and induce lysophagy via different pathways^84,86^. For example, Gal8 directly binds to LC3 on the phagophore through autophagy receptor NDP52, in turn driving damaged lysosomes toward an autophagy pathway^84^. In addition to galectins, other molecules can be ubiquitinated on the damaged lysosomes^85^. Skp1/CUL1/F-box (SCF) is a ligase complex, and F-box protein is the receptor of the substrate of the SCF ubiquitin-ligase complex. When lysosomes were disrupted by LLOME treatment, SCF^FBXO27^ was rapidly recruited to the broken lysosomes and ubiquitinated LAMP-2 to activate lysophagy^85^. Given that lysophagy is a normal lysosome-dependent pathway that eliminates damaged lysosomes, the timely reestablishment of lysosomal numbers is crucial for the maintenance of lysosomal function.

### Lysosomal regeneration

Lysosomal biogenesis requires the integration of both cellular endocytosis and protein biosynthesis, which is realized through the formation of the membrane structure of lysosomes and the provision of lysosomal proteins, respectively^3,87–89^. The lysosomal membrane is constituted by both the primary lysosomes secreted by the Golgi and vesicles from the plasma membrane and the endocytic pathway^3^. Newly synthesized proteins targeted to lysosomes can be transported either directly into the lysosome through the trans-Golgi network depending on receptors, including mannose-6-phosphate receptor, sortilin, and lysosomal integral membrane protein type 2^87,88^, or indirectly ingested into the lumen by endocytosis^89^.

Under physiological conditions, the number of lysosomes is continuously maintained by dynamic homeostasis balanced between formation and degradation^83^. Lysosomal regeneration is initiated when lysosomes are lost^83^. Bioinformatics and functional genome analysis have revealed that the promoter regions of many lysosomal genes contain one or more repeats of the 10-base pair motif (GTCACGTGAC), known for coordinating lysosomal expression and regulation (CLEAR) elements^90,91^. TFEB, a member of the MiT/TFE family, has been found to directly bind with the CLEAR element and further promote lysosomal renewal^92^. Further study confirmed that TFEB contributed to the expression levels of a large number of genes involved in lysosomal function, including exocytosis, phagocytosis, endocytosis, and autophagy^90^. For example, the inhibition of mTOR enabled TFEB dephosphorylation and translocation the later increased the expression of genes encoding lysosomal proteins, such as V-ATPases, lysosomal transmembrane proteins, and hydrolases^93^.

In general, endolysosome damage is initiated rapidly in response to lysosomal damage, and multiple processes are involved in the repair of damaged lysosomes and cell survival. Thus, it is of great significance to identify the molecular mechanisms of LQC, and its relationship with the occurrence and development of clinical diseases.

## Lysosomal damage and cell fate

**Fig. 3:**
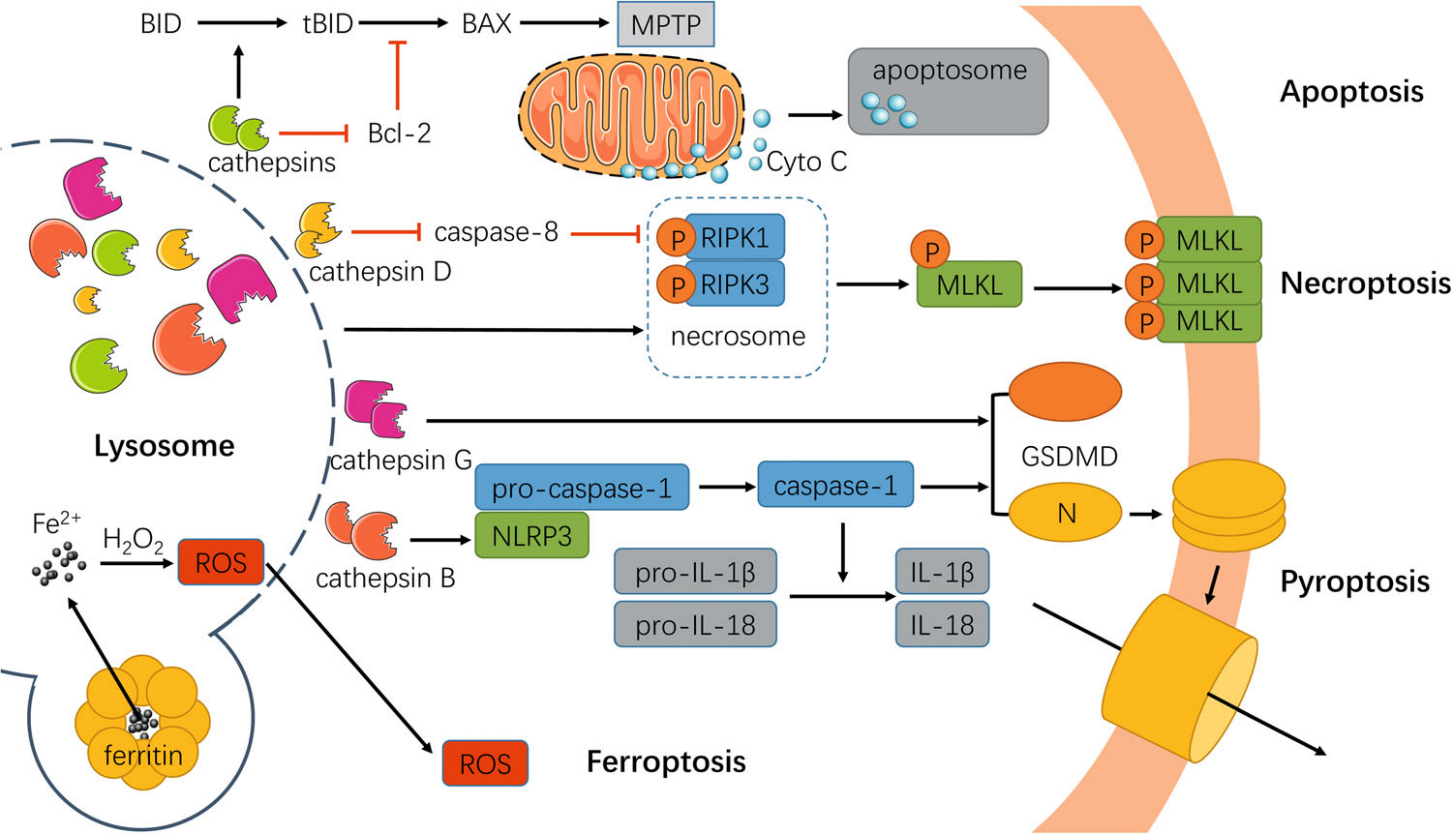
Lysosomal damage and cell fate. Apoptosis appears to be triggered by lysosomal damage in a mitochondria-dependent manner. Cathepsins are released from damaged lysosomes and cleave BID into tBid, in turn promoting the oligomorphism of BAX, which can be further enhanced by cathepsin-induced degradation of Bcl-2. Then, BAX is transferred to the mitochondrial outer membrane, causing excessive formation of the MPTP. Through the MPTP, cyto C is released into the cytoplasm and promotes the formation of apoptosomes, further inducing apoptosis. Necroptosis is stimulated by the inhibition of lysosomal function, resulting in significant accumulation of necrosome components (RIPK1 and RIPK3) and hydrolyzed caspase-8 by the release of cathepsin D. Necroptosis executor (MLKL) is phosphorylated by necrosomes and translocated to the cell membrane or organelle membrane, thereby leading to necroptosis. Pyroptosis is induced by damaged lysosomes through the cleavage of GSDMD into GSDMD-N by the release of cathepsin G, and activating NLRP3 and caspase-1 by the release of cathepsin B. Subsequently, pyroptosis leads to cell perforation and the massive release of IL-1β and IL-18. Additionally, ferroptosis can be exacerbated by the release of ROS produced by Fe^2+^ and H_2_O_2_ from damaged lysosomes.

Persistent lysosomal damage poses a serious threat to cell fate. Lysosomal injury-associated cell death was first identified by de Duve, who defined the lysosome as a “suicide bag”^94^. In fact, the degree of lysosomal damage is the determinant of the type of cell death. Early studies suggested that moderate lysosomal damage can induce cell apoptosis, while extensive damage resulted in a substantial number of cells undergoing irreversible necrosis. Accumulating evidence has indicated that lysosomal damage is closely related to the development of apoptosis^6,95^, necroptosis^8,10^, pyroptosis^11^, and ferroptosis^13,96^ (Fig. 3).

### Apoptosis

Apoptosis is defined as programmed cell death. Currently, two main pathways are widely accepted as mediating apoptosis, namely exogenous apoptosis via cell surface death receptors, and intrinsic apoptosis dependent on mitochondria involvement^97^. Intrinsic apoptosis is initiated under exposure to internal stimuli, such as growth factor deprivation, hypoxia, DNA damage, oxidative stress, and calcium overload^98^. In this pathway, apoptosis is trigger by the release of cytochrome C (cyto C) from mitochondria, which further induces the activation of caspase cascades^99^. Lysosomal damage triggers apoptosis through the endogenous pathway. When LMP or lysosomal rupture occurs, cathepsins are released from lysosomes and cleave BH3-interacting domain death agonist (BID) into tBid fragments, which promote the oligomorphism of B-cell lymphoma-2 (Bcl-2)-associated X protein^6^. Then, the oligomorphic complex is transferred to the outer mitochondrial membrane, causing the excessive formation of the mitochondrial permeability transition pore (MPTP)^6^. Cyto C is released through the MPTP into the cytoplasm, where it augments the formation of apoptosomes^6,7^. In summary, the leakage of lysosomal enzymes from damaged lysosomes is critical for apoptosis in the mitochondrial-dependent pathway. Interestingly, the release of H_2_O_2_ from damaged mitochondria also accounts for the fragmentation of lysosomal membranes^100^. In addition, cathepsins are capable of degrading Bcl-2, thereby leading to exacerbated cellular apoptosis^5^. Timely recognition and prompt interference of lysosomal damage are efficient measures for preventing cellular apoptosis. For example, the apoptosis of HepG2 cells can be significantly inhibited by specific cathepsin B inhibitors^95,101^. The inhibition of cathepsin B and cathepsin L by interfering with the mitochondrial pathway markedly protected astrocytes from apoptosis^102^.

### Necroptosis

The traditional view suggests that necrosis is a nonprogrammed or “accidental” cell death. However, it has been demonstrated that necrosis can be driven and regulated by certain molecular mechanisms, similar to apoptosis, in a process called necroptosis^103^. The morphological characteristics of necroptosis are similar to those of necrosis, but its initiation is noncaspase-dependent^104,105^. The currently accepted concept suggests that necroptosis is mediated by receptor-interacting protein kinase (RIPK) and mixed-lineage kinase domain-like protein (MLKL). Pasparakis et al.^106^ found that tumor necrosis factor (TNF)-α was capable of activating RIPK1, promoting the phosphorylation of RIPK3, and thus forming RIPK1–RIPK3 complex, named a necrosome. MLKL was then phosphorylated by the necrosome and translocated to the cell membrane or organelle membrane, thus leading to necroptosis^107^. It was recently revealed that the inhibition of lysosomal function after traumatic spinal cord injury led to the significant accumulation of RIPK1, RIPK3, and MLKL in neurons, which was alleviated by the increased activation of autophagy-lysosome pathways^8^. Caspase-8 is a major negative regulator of necroptosis by interfering with RIPK activation and preventing the formation of necrosomes^9^. The release of cathepsin D induced necroptosis through the proteolysis of caspase-8 and promotion of RIPK1 activation, implying that lysosomal dysfunction might be crucially involved in the onset of necroptosis^9^.

### Pyroptosis

Pyroptosis is characterized by both induction of a proinflammatory response and programmed cell death, which is realized mainly through the activation of caspase-1 and the proteolysis of GSDMD, accompanied by cell perforation and excessive release of IL-1β and IL-18^108^. The cathepsins released from damaged lysosomes play important roles in the process of pyroptosis. It has been shown that cathepsin G effectively induces the development of pyroptosis by cleaving GSDMD into GSDMD-N^10^. The activation of caspase-1 primarily depends on NOD-like receptors that recruit caspase-1 and further induce caspase-1 activation by binding with NOD-like receptor protein (NLRP)3 inflammasomes^109^. In addition, released cathepsin B can activates NLRP3 and caspase-1 by binding to the leucine-rich repeat domain of NLRP3, hinting to the critical involvement of lysosomal damage in cellular pyroptosis^11^.

### Ferroptosis

Ferroptosis is a form of regulated cell death characterized by excessive intracellular accumulation of iron-dependent lipid hydroperoxides^110^. Either the overproduction of the Fe^2+^ that is involved in the formation of lipid hydroperoxides through the Fenton reaction^111^ or the inactivation of glutathione peroxidase 4, which is programmed to degrade lipid hydroperoxides, leads to intracellular lipid hydroperoxide accumulation to a lethal level, ultimately triggering ferroptosis^112^. An imbalance between intracellular reactive oxygen species (ROS) production and clearance is among the major causes of cellular ferroptosis^110^. The lysosome is one of the main storage sites for iron and essential for maintaining the levels of intracellular ROS and iron. Upon exposure to H_2_O_2_, the free iron in lysosomes increases the production of ROS, which can then be released through an unstable lysosomal membrane to trigger the activation of cell death pathways^12^. Most of the iron is in the form suitable for binding with ferritin in the cytoplasm, which can be degraded by autophagy-lysosome pathway, causing the release of active iron^113^. The inhibition of the autophagy–lysosome pathway was reportedly capable of slowing drug-induced ferroptosis, indicating that the autophagy–lysosome pathway appeared to be associated with the occurrence of ferroptosis^13^. However, ferroptosis can also be restricted by inhibiting lysosomal function through the downregulation of cathepsin or V-ATPase activity^96^.

## Lysosomes and various diseases

A growing body of evidence indicates that the structural and functional stability of lysosomes are essential for maintaining cellular homeostasis^2,3^. Lysosomal dysfunction might play an important role in the development of various diseases, including tumors^14–16^, neurodegenerative diseases^17–19^, inflammatory diseases^20–22^, and cardiovascular diseases^23–25^ (Table 1).

**Table 1:** Lysosome as the therapeutic target in various diseases.

Diseases		Lysosome associated alterations	Consequenses	Intervention and therapy
Tumor	PDA	Upregulation of lysosomal and autophagic function by activation of TFEB	Catabolic hyperactivity and prone to invasion and progression	ATG5 or ATG7 knock out, hydroxychloroquine and its derivatives alone or in combination with standard chemotherapy drugs
	Breast cancer	Overexpression of cathepsin B		Downregulating the shRNA of cathepsin B
Neurodegenerative diseases	GD	Abnormal transport of glucosinolates to lysosomes	Neurological dysfunction	Enzyme replacement therapy (injecting glucosinolidase or its structural analog)
	HSP	Damaged lysosomal biogenes caused by mutations in SPG11 and SPG15 genes, intracellular accumulation of gangliosides	Progressive spasm of the lower limbs caused by axial mutation of upper motor neurons	Alleviating the degradation pressure of lysosomes and improving lysosomal function
	AD	Increased lysosomal pH and impaired lysosomal proteolytic function	AD-like pathologic change	
Inflammatory diseases	Acute hyperuricemia	Lysosomal membrane destroyed and lysosomal rupture by uric acid crystals	Renal inflammation	
	AP	Activation of cell death pathway by cathepsin B leaked into the cytoplasm	Pancreatic injury mainly in the form of cellular apoptosis and necrosis	
	Sepsis	Upregulation of the key genes of lysosomes	Immunosuppression and multiple organ dysfunction	A variety of drugs used to reverse the immunosuppression of sepsis have been proved to be related to lysosomal and autophagic function
Cardiovascular diseases	AS	Decrease of LAL activity by excessive free cholesterol accumulated in lysosomes	Accumulation of cholesterol esters in lysosomes and cytoplasm in foam cells	Infusion of recombinant human LAL
	Danon disease	Deletion of LAMP2 gene	Lysosomal and autophagosome fusion disorders, massive autophagosome deposition in the cytoplasm of myocardium and skeletal muscle	Enhancing lysosomal function by TFEB overexpression

*AD* Alzheimer’s disease, *AP* acute pancreatitis, *AS* atherosclerosis, *ATG* autophagy-related gene, *GD* Gaucher disease, *HSP* hereditary spastic paraplegias, *LAL* lysosomal acid lipase, *LAMP2* lysosome-associated membrane protein 2, *PDA* pancreatic ductal adenocarcinoma, *SPG* spastic paraplegia, *TFEB* transcription factor EB.

### Tumors

Catabolic hyperactivity is one of the main characteristics of tumor cells, and it is the major reason for tumor progression and metastasis^14^. The rapid proliferation of tumor cells is guaranteed by high synthesis rates, which largely depend on the degradation and recycling of cellular components^28^. The autophagy–lysosome system is fully activated to meet the metabolic needs of tumor cells^28^. For example, TFEB was found to maintain high levels of autophagic activity by upregulating lysosomal function in pancreatic ductal adenocarcinoma (PDA)^15^. The knockout of autophagy-related gene (ATG) 5 or ATG7 and the inhibition of lysosomal function significantly diminished the invasion and progression of PDA, suggesting that downregulation of lysosomal function is associated with inhibited tumor progression^114^. A number of clinical trials have been conducted to treat tumors by targeting lysosomes, such as through the anti-breast-cancer effects of saponin D, which blocks autophagosome–lysosome fusion^115^. Therefore, disturbing the autophagy–lysosome function is efficient for delaying the occurrence and development of tumors, and it is gradually becoming a promising target for tumor treatment.

In addition, cathepsin B is considered to be involved in proteolytic cascade destruction of the extracellular matrix, which further remodels the tumor environment by enabling angiogenesis, tumor migration, invasion, and even metastasis^16^. The overexpression of cathepsin B promoted the invasiveness and progression of breast cancer cells^116,117^, while downregulation of cathepsin B function by shRNA significantly reduced bone metastasis in mice breast cancer models^118^. The abnormal distribution of lysosomes is critically involved in carcinogenesis. Under normal conditions, lysosomes are mainly located in the peripheral and perinuclear regions of eukaryocytes^119^. During the process of carcinogenesis, lysosomes significantly transfer from the perinuclear region to the peripheral cytoplasm, which led to enhanced lysosomal exocytosis^28^. Furthermore, lysosomal exocytosis of hydrolytic enzymes promoted the invasion of tumor cells and degradation of the extracellular matrix, thereby contributing to cancer progression^120^. Thus, the regulation of lysosomal distribution should be taken seriously in anticancer therapy.

### Neurodegenerative diseases

Neurons cannot dilute damaged organelles and cellular waste through cell division, implying the great need for an efficient intracellular clearance system^121,122^. Strikingly, lysosomes are essential for the functional maintenance and survival of neurons. An increasing number of studies have documented that failed lysosomal function is closely related to the occurrence and development of neurodegenerative diseases, including Gaucher disease, hereditary spastic paraplegias, and Alzheimer’s disease (AD)^18,123–125^. Gaucher disease is a rare autosomal recessive genetic disease that is caused by the excessive accumulation of glucose neuraminidase in macrophages because of the dysfunction and abnormal lysosomal transport of glucosaminidase^123^. The clinical manifestations of neurological Gaucher disease vary by subtype and manifest different symptoms^123^. Enzyme replacement therapy has achieved remarkable curative effects by enhancing the decomposition of glycosphingolipids in lysosomes, relieving the pressure on lysosomes, and improving lysosomal function^123^. Hereditary spastic paraplegias is a highly heterogeneous autosomal recessive hereditary disease most commonly caused by mutations in SPG11 and SPG15^124^. Lack of these two proteins can prevent lysosomal biogenesis and autophagosome maturity, leading to the ganglioside accumulation and neuronal death^124^. A recent study showed that alleviating ganglioside accumulation can significantly reduce the number of neuron deaths and thus improve the disease phenotype in zebrafish models with the SPG11 mutation^124^. Thus, alleviating the degradation pressure on lysosomes and improving lysosomal function are effective strategies for the management of neurodegenerative diseases. Additionally, lysosomal acidification is a major factor in the activity of most lysosomal hydrolytic enzymes and the regulation of lysosomal signals^19^. For example, the mutant presenilin 1, one of the main causes of familial AD, was found to cause a dysfunction in lysosomal acidification, leading to elevated lysosomal pH values^125^, lysosomal proteolysis injury, and AD-like pathologic alterations^126^. Similarly, two subtypes of early-onset Parkinson’s disease (PD), XPDS and Kufor-Rakeb syndrome, are both related to disorders of lysosomal acidification^127,128^, indicating that lysosomal deacidification accelerates the development of PD-like neuropathology. Some nerve agents that aggravate PD and PD-like symptoms have been shown to disturb pH values in lysosomes^129,130^.

### Inflammatory diseases

Inflammation, deemed a protective response to pathogens, irritation, and injury^131^, can effectively remove invading pathogens and maintain tissue integrity by releasing inflammatory mediators, inducing inflammatory cell migration and aggregation^132^. However, excessive or sustained inflammation results in the destruction of tissues, followed by the development of inflammatory diseases. It has been generally accepted that lysosomes are critical to the initiation and recession of inflammatory responses^37^. In hyperuricemia, uric acid is supersaturated in urine and forms uric acid crystals, which are transported to lysosomes by the endocytosis of renal tubular epithelial cells, destroying the lysosomal membrane and causing lysosomal rupture, in turn, resulting in renal inflammation^20^. It was found that renal tubular epithelial cells with the Atg5 gene knocked out failed to isolate the damaged lysosomes, which caused irreversible cell damage in mouse models of acute hyperuricemic nephritis^83^. Similarly, the coexistence of lysosomes and digestive enzymes in pancreatic acinar cells is considered an early event in acute pancreatitis^21^. Activation of trypsin induces the leakage of lysosomes and the release of cathepsin B into the cytoplasm, which activates the cell death pathway and finally causes pancreatic injury mainly in the forms of cellular apoptosis and necrosis^21^. Taken together, these studies show that maintaining the integrity of the lysosomal structure and function or inhibiting the activity of cathepsin B, may be promising therapeutic targets for acute pancreatitis.

In the context of sepsis, stimulation of autophagy can lead not only to the removal of damaged organelles and redundant proteins, but also to the removal of invading pathogenic microorganisms, a process that has become a potential target for improving the survival and prognosis of septic complications^133^. The activity of autophagy increases significantly at the early stage of sepsis, which shows obvious advantages by promoting pathogen clearance, attenuating cell apoptosis, and modulating inflammation^134^. However, autophagy is remarkably inhibited during persistent exposure to septic challenge, and enhancing autophagic activity can significantly reduce the immunosuppression and organ dysfunction secondary to severe sepsis^135,136^. It was recently reported that a variety of drugs used to reverse sepsis-induced immunosuppression were related to induced autophagy activation^136,137^. In addition, mRNA expression of key genes in lysosomes, including acid hydrolases (such as cathepsin A/D), LAMP-1 and LAMP-2, was significantly upregulated following sepsis, hinting that overloaded lysosomal function might be an early marker for poor outcomes for patients with sepsis^22^.

### Cardiovascular diseases

Given the critical involvement of autophagy in cardiovascular diseases, such as myocardial ischemic and reperfusion injury, cardiomyopathy, myocardial infarction, and heart failure^138–142^, the roles of lysosomes as the terminal sites of the autophagic process are indispensable. In myocardial ischemia and reperfusion injury, cardiomyocyte death can be caused by lysosomal consumption, which manifests as a decrease in the number of lysosomes and the downregulated expression of LAMP-1^139^. Both the enhancement of lysosomal function and promotion of autophagy were reportedly beneficial by alleviating cardiomyocyte death after myocardial ischemia and reperfusion injury^139,143^. In proteotoxic cardiomyopathy, cardiomyocytes showed impaired autophagic flux and reduced lysosome abundance; however, adeno-associated virus-TFEB transduction was sufficient to reverse cardiomyopathy pathology in a preclinical study^142^. Both dysfunctional lysosomes and impaired autophagic processes might contribute to the development of myocardial infarction and heart failure, which may be improved by enhancing autophagic activity^140,141^. Lysosomes are critically involved in the development and progression of cardiovascular diseases in autophagy-independent ways.

Atherosclerosis refers to lipid deposition (mainly cholesterol and cholesteryl ester) in the intima and subintima of arteries. Lipids induce the dysfunction of lysosomes in mice macrophages, as shown by an increase in lysosomal pH, decrease in proteolytic ability, and destruction of membrane integrity^24^. Lysosomal acid lipase is the main enzyme that hydrolyzes low-density lipoproteins and the modified forms of low-density lipoproteins, playing a pivotal role in lipid decomposition and prevention of excessive lipid accumulation^144^. Upon the development of atherosclerosis, excessive accumulation of free cholesterol leads to the suppression of lysosomal acid lipase activity and the accumulation of cholesterol esters in lysosomes^145^. In addition, deficiency of primary LAMP-2 is associated with the occurrence of Danon disease^25^, as evidenced by the pathological characteristics of massive autophagosome deposition in cardiomyocytes and skeletal muscle cells^146,147^. Similarly, mice with LAMP-2 knocked out show impaired autophagy and autophagosomes aggregation in cardiomyocytes, resulting in cardiac defects^148^. Taken together, studies show the critical involvement of lysosomes in the occurrence and development of cardiovascular diseases, which has been confirmed by their decreased number and significant dysfunction, as well as an impaired autophagic process, leading to aberrant cellular responses and even cell death. While upregulation of the autophagy–lysosome pathway or reduction in lysosomal stress may alleviate the progression of cardiomyocyte damage and prevent cell death, providing novel insights into disease therapy.

## Conclusions and perspectives

In-depth understanding of lysosomes has shown that they are not simple “digestive centers” but are main metabolic centers and signal hubs. The degradation function of lysosomes has been adequately studied for decades, but studies on the roles of lysosomes as cellular sensors and signal hubs are in an initial state. Although the mechanism of amino acid sensing has been clarified, the impacts of other nutrients on lysosomal signaling are largely unclear. As LQC is critically involved in maintaining cell viability and function, the general aspects of LQC have been gradually emphasized and revealed; nevertheless, some details remain unclear. For example, the interaction between components of the ESCRT complex and their precise mechanisms in lysosomal repair are largely unknown. Based on the current evidence, the selective degradation process of lysosomes via lysophagy is thought to be facilitated by the recognition of lysosomal proteins exposed to cytoplasm after rupture. Therefore, similar to its significance in organelle-specific autophagy, the importance of discovering specific lysosome-anchored receptors is self-evident. The critical involvement of lysosomal damage in different pathways of cell death has been widely studied, but the current focus is placed on the release of cathepsin-based contents caused by structural damage. Few studies have addressed cell death induced by the release of ions, other proteases, and/or the dysfunction of the degradation or signal sensing roles of lysosomes.

Lysosomes play key roles in the development and progression of various human diseases, mainly through the alteration of their degradation capacity and the release of their contents, resulting in catabolic hyperactivity in tumors, accumulation of abnormal substances that causes neurodegenerative and cardiovascular diseases, and excessive inflammatory responses in inflammatory diseases. Therapies targeting lysosomes might exert beneficial effects for disease remission. For example, the inhibition of lysosomal function in cancer cells can restrain tumor metabolism, thereby alleviating tumor progression. In addition, the enhancement of lysosomal function and lysosomal enzyme replacement can accelerate the clearance of accumulated pathogenesis-related proteins in neurodegenerative and cardiovascular diseases. Similarly, the protection of lysosomes from structural damage and functional impairment can remarkably alleviate the progression of inflammatory diseases. In view of the indispensable role of LQC in lysosomal structure and function maintenance, LQC might also serve as a potential therapeutic target for treating human diseases.

## References

[1] de Duve C (2005). The lysosome turns fifty. Nat. Cell Biol..

[2] Appelqvist H, Wäster P, Kågedal K, Öllinger K (2013). The lysosome: from waste bag to potential therapeutic target. J. Mol. Cell Biol..

[3] Perera RM, Zoncu R (2016). The lysosome as a regulatory hub. Annu. Rev. Cell Dev. Biol..

[4] Carroll B, Dunlop EA (2017). The lysosome: a crucial hub for AMPK and mTORC1 signalling. Biochem. J..

[5] Česen MH, Pegan K, Spes A, Turk B (2012). Lysosomal pathways to cell death and their therapeutic applications. Exp. Cell Res..

[6] Zhu X (2017). Mastocarcinoma therapy synergistically promoted by lysosome dependent apoptosis specifically evoked by 5-Fu@nanogel system with passive targeting and pH activatable dual function. J. Control Release.

[7] Taylor RC, Cullen SP, Martin SJ (2008). Apoptosis: controlled demolition at the cellular level. Nat. Rev. Mol. Cell Biol..

[8] Liu S (2018). Lysosomal damage after spinal cord injury causes accumulation of RIPK1 and RIPK3 proteins and potentiation of necroptosis. Cell Death Dis..

[9] Zou J (2013). Poly IC triggers a cathepsin D- and IPS-1-dependent pathway to enhance cytokine production and mediate dendritic cell necroptosis. Immunity.

[10] Burgener SS (2019). Cathepsin G inhibition by serpinb1 and serpinb6 prevents programmed necrosis in neutrophils and monocytes and reduces GSDMD-driven inflammation. Cell Rep..

[11] Bruchard M (2013). Chemotherapy-triggered cathepsin B release in myeloid-derived suppressor cells activates the NLRP3 inflammasome and promotes tumor growth. Nat. Med..

[12] Ostenfeld MS (2005). Effective tumor cell death by sigma-2 receptor ligand siramesine involves lysosomal leakage and oxidative stress. Cancer Res..

[13] Torii S (2016). An essential role for functional lysosomes in ferroptosis of cancer cells. Biochem. J..

[14] Piao S, Amaravadi RK (2016). Targeting the lysosome in cancer. Ann. N. Y. Acad. Sci..

[15] Perera RM (2015). Transcriptional control of autophagy-lysosome function drives pancreatic cancer metabolism. Nature.

[16] Schmitz J (2019). Cathepsin B: active site mapping with peptidic substrates and inhibitors. Bioorg. Med. Chem..

[17] Lie PPY, Nixon RA (2019). Lysosome trafficking and signaling in health and neurodegenerative diseases. Neurobiol. Dis..

[18] Thelen AM, Zoncu R (2017). Emerging roles for the lysosome in lipid metabolism. Trends Cell Biol..

[19] Colacurcio DJ, Nixon RA (2016). Disorders of lysosomal acidification—the emerging role of v-ATPase in aging and neurodegenerative disease. Ageing Res. Rev..

[20] Kimura T, Isaka Y, Yoshimori T (2017). Autophagy and kidney inflammation. Autophagy.

[21] Talukdar R (2016). Release of cathepsin B in cytosol causes cell death in acute pancreatitis. Gastroenterology.

[22] Ma J (2015). Lysosome and cytoskeleton pathways are robustly enriched in the blood of septic patients: a meta-analysis of transcriptomic data. Mediat. Inflamm..

[23] Liu CL (2018). Cysteine protease cathepsins in cardiovascular disease: from basic research to clinical trials. Nat. Rev. Cardiol..

[24] Emanuel R (2014). Induction of lysosomal biogenesis in atherosclerotic macrophages can rescue lipid-induced lysosomal dysfunction and downstream sequelae. Arterioscler. Thromb. Vasc. Biol..

[25] Nishino I (2000). Primary LAMP-2 deficiency causes X-linked vacuolar cardiomyopathy and myopathy (Danon disease). Nature.

[26] Papadopoulos C, Meyer H (2017). Detection and clearance of damaged lysosomes by the endo-lysosomal damage response and lysophagy. Curr. Biol..

[27] Mindell JA (2012). Lysosomal acidification mechanisms. Annu. Rev. Physiol..

[28] Pu J, Guardia CM, Keren-Kaplan T, Bonifacino JS (2016). Mechanisms and functions of lysosome positioning. J. Cell Sci..

[29] Settembre C, Fraldi A, Medina DL, Ballabio A (2013). Signals from the lysosome: a control centre for cellular clearance and energy metabolism. Nat. Rev. Mol. Cell Biol..

[30] Saxton RA (2016). Structural basis for leucine sensing by the Sestrin2-mTORC1 pathway. Science.

[31] Wyant GA (2017). mTORC1 activator SLC38A9 is required to efflux essential amino acids from lysosomes and use protein as a nutrient. Cell.

[32] Li P, Gu M, Xu H (2019). Lysosomal ion channels as decoders of cellular signals. Trends Biochem. Sci..

[33] Xiong J, Zhu MX (2016). Regulation of lysosomal ion homeostasis by channels and transporters. Sci. China Life Sci..

[34] Luzio JP, Parkinson MD, Gray SR, Bright NA (2009). The delivery of endocytosed cargo to lysosomes. Biochem. Soc. Trans..

[35] Kaur J, Debnath J (2015). Autophagy at the crossroads of catabolism and anabolism. Nat. Rev. Mol. Cell Biol..

[36] Singh R, Cuervo AM (2011). Autophagy in the cellular energetic balance. Cell Metab..

[37] Inpanathan S, Botelho RJ (2019). The lysosome signaling platform: adapting with the times. Front. Cell Dev. Biol..

[38] Savini M, Zhao Q, Wang MC (2019). Lysosomes: signaling hubs for metabolic sensing and longevity. Trends Cell Biol..

[39] Lamming DW, Bar-Peled L (2019). Lysosome: the metabolic signaling hub. Traffic.

[40] Uribe-Querol E, Rosales C (2017). Control of phagocytosis by microbial pathogens. Front. Immunol..

[41] Lim JJ, Grinstein S, Roth Z (2017). Diversity and versatility of phagocytosis: roles in innate immunity, tissue remodeling, and homeostasis. Front. Cell Infect. Microbiol..

[42] Rosales C, Uribe-Querol E (2017). Phagocytosis: a fundamental process in immunity. Biomed. Res. Int..

[43] Gordon S (2016). Phagocytosis: an immunobiologic process. Immunity.

[44] Fairn GD, Grinstein S (2012). How nascent phagosomes mature to become phagolysosomes. Trends Immunol..

[45] Feng Y, He D, Yao Z, Klionsky DJ (2014). The machinery of macroautophagy. Cell Res..

[46] Li WW, Li J, Bao JK (2012). Microautophagy: lesser-known self-eating. Cell Mol. Life Sci..

[47] Kaushik S, Cuervo AM (2018). The coming of age of chaperone-mediated autophagy. Nat. Rev. Mol. Cell Biol..

[48] Chiang HL, Terlecky SR, Plant CP, Dice JF (1989). A role for a 70-kilodalton heat shock protein in lysosomal degradation of intracellular proteins. Science.

[49] Bandyopadhyay U, Kaushik S, Varticovski L, Cuervo AM (2008). The chaperone-mediated autophagy receptor organizes in dynamic protein complexes at the lysosomal membrane. Mol. Cell Biol..

[50] Rout AK, Strub MP, Piszczek G, Tjandra N (2014). Structure of transmembrane domain of lysosome-associated membrane protein type 2a (LAMP-2A) reveals key features for substrate specificity in chaperone-mediated autophagy. J. Biol. Chem..

[51] Yim WW, Mizushima N (2020). Lysosome biology in autophagy. Cell Discov..

[52] Salvador N, Aguado C, Horst M, Knecht E (2000). Import of a cytosolic protein into lysosomes by chaperone-mediated autophagy depends on its folding state. J. Biol. Chem..

[53] Agarraberes FA, Dice JF (2001). A molecular chaperone complex at the lysosomal membrane is required for protein translocation. J. Cell Sci..

[54] Mizushima N, Komatsu M (2011). Autophagy: renovation of cells and tissues. Cell.

[55] Yao, R. Q., Ren, C., Xia, Z. F. & Yao, Y. M. Organelle-specific autophagy in inflammatory diseases: a potential therapeutic target underlying the quality control of multiple organelles. *Autophagy*. 10.1080/15548627.2020.1725377 (2020). 10.1080/15548627.2020.1725377 PMC8007140 32048886

[56] Shaid S, Brandts CH, Serve H, Dikic I (2013). Ubiquitination and selective autophagy. Cell Death Differ..

[57] Weidberg H, Shvets E, Elazar Z (2011). Biogenesis and cargo selectivity of autophagosomes. Annu. Rev. Biochem..

[58] Gatica D, Lahiri V, Klionsky DJ (2018). Cargo recognition and degradation by selective autophagy. Nat. Cell Biol..

[59] Hesketh GG, Wartosch L, Davis LJ, Bright NA, Luzio JP (2018). The lysosome and intracellular signalling. Prog. Mol. Subcell. Biol..

[60] Saxton RA, Sabatini DM (2017). mTOR signaling in growth, metabolism, and disease. Cell.

[61] Sancak Y (2010). Ragulator-Rag complex targets mTORC1 to the lysosomal surface and is necessary for its activation by amino acids. Cell.

[62] Wang S (2015). Lysosomal amino acid transporter SLC38A9 signals arginine sufficiency to mTORC1. Science.

[63] Bar-Peled L, Schweitzer LD, Zoncu R, Sabatini DM (2012). Ragulator is a GEF for the rag GTPases that signal amino acid levels to mTORC1. Cell.

[64] Zoncu R (2011). mTORC1 senses lysosomal amino acids through an inside-out mechanism that requires the vacuolar H(+)-ATPase. Science.

[65] Peng M, Yin N, Li MO (2017). SZT2 dictates GATOR control of mTORC1 signalling. Nature.

[66] Parmigiani A (2014). Sestrins inhibit mTORC1 kinase activation through the GATOR complex. Cell Rep..

[67] Long X, Lin Y, Ortiz-Vega S, Yonezawa K, Avruch J (2005). Rheb binds and regulates the mTOR kinase. Curr. Biol..

[68] Ben-Sahra I, Manning BD (2017). mTORC1 signaling and the metabolic control of cell growth. Curr. Opin. Cell Biol..

[69] Iadevaia V, Liu R, Proud CG (2014). mTORC1 signaling controls multiple steps in ribosome biogenesis. Semin. Cell Dev. Biol..

[70] Martina JA, Chen Y, Gucek M, Puertollano R (2012). mTORC1 functions as a transcriptional regulator of autophagy by preventing nuclear transport of TFEB. Autophagy.

[71] Zhang T (2017). Structural basis for Ragulator functioning as a scaffold in membrane-anchoring of Rag GTPases and mTORC1. Nat. Commun..

[72] Hardie DG, Ross FA, Hawley SA (2012). AMPK: a nutrient and energy sensor that maintains energy homeostasis. Nat. Rev. Mol. Cell Biol..

[73] Papadopoulos C, Kravic B, Meyer H (2020). Repair or lysophagy: dealing with damaged lysosomes. J. Mol. Biol..

[74] Bove J (2014). BAX channel activity mediates lysosomal disruption linked to Parkinson disease. Autophagy.

[75] Repnik U, Hafner Česen M, Turk B (2014). Lysosomal membrane permeabilization in cell death: concepts and challenges. Mitochondrion.

[76] Serrano-Puebla A, Boya P (2016). Lysosomal membrane permeabilization in cell death: new evidence and implications for health and disease. Ann. N. Y. Acad. Sci..

[77] Gomez-Sintes R, Ledesma MD, Boya P (2016). Lysosomal cell death mechanisms in aging. Ageing Res. Rev..

[78] Kirkegaard T (2010). Hsp70 stabilizes lysosomes and reverts Niemann-Pick disease-associated lysosomal pathology. Nature.

[79] Petersen NH, Kirkegaard T, Olsen OD, Jaattela M (2010). Connecting Hsp70, sphingolipid metabolism and lysosomal stability. Cell Cycle.

[80] Skowyra, M. L., Schlesinger, P. H., Naismith, T. V. & Hanson, P. I. Triggered recruitment of ESCRT machinery promotes endolysosomal repair. *Science* **360** (2018). 10.1126/science.aar5078 PMC6195421 29622626

[81] Radulovic, M. et al. ESCRT-mediated lysosome repair precedes lysophagy and promotes cell survival. *Embo J.*10.15252/embj.201899753 (2018). 10.15252/embj.201899753 PMC6213280 30314966

[82] Christ L, Raiborg C, Wenzel EM, Campsteijn C, Stenmark H (2017). Cellular functions and molecular mechanisms of the ESCRT membrane-scission machinery. Trends Biochem. Sci..

[83] Maejima I (2013). Autophagy sequesters damaged lysosomes to control lysosomal biogenesis and kidney injury. Embo J..

[84] Thurston TL, Wandel MP, von Muhlinen N, Foeglein A, Randow F (2012). Galectin 8 targets damaged vesicles for autophagy to defend cells against bacterial invasion. Nature.

[85] Yoshida Y (2017). Ubiquitination of exposed glycoproteins by SCF(FBXO27) directs damaged lysosomes for autophagy. Proc. Natl Acad. Sci. USA.

[86] Chauhan S (2016). TRIMs and Galectins globally cooperate and TRIM16 and Galectin-3 co-direct autophagy in endomembrane damage homeostasis. Dev. Cell.

[87] Braulke T, Bonifacino JS (2009). Sorting of lysosomal proteins. Biochim Biophys. Acta.

[88] Reczek D (2007). LIMP-2 is a receptor for lysosomal mannose-6-phosphate-independent targeting of beta-glucocerebrosidase. Cell.

[89] Saftig P, Klumperman J (2009). Lysosome biogenesis and lysosomal membrane proteins: trafficking meets function. Nat. Rev. Mol. Cell Biol..

[90] Palmieri M (2011). Characterization of the CLEAR network reveals an integrated control of cellular clearance pathways. Hum. Mol. Genet..

[91] Sardiello M (2009). A gene network regulating lysosomal biogenesis and function. Science.

[92] Napolitano G, Ballabio A (2016). TFEB at a glance. J. Cell Sci..

[93] Roczniak-Ferguson A (2012). The transcription factor TFEB links mTORC1 signaling to transcriptional control of lysosome homeostasis. Sci. Signal..

[94] de Duve C (1983). Lysosomes revisited. Eur. J. Biochem..

[95] Gao C (2014). Tacrine induces apoptosis through lysosome- and mitochondria-dependent pathway in HepG2 cells. Toxicol. in Vitro.

[96] Gao H (2018). Ferroptosis is a lysosomal cell death process. Biochem. Biophys. Res. Commun..

[97] Hengartner MO (2000). The biochemistry of apoptosis. Nature.

[98] Tait SW, Green DR (2010). Mitochondria and cell death: outer membrane permeabilization and beyond. Nat. Rev. Mol. Cell Biol..

[99] Riedl SJ, Salvesen GS (2007). The apoptosome: signalling platform of cell death. Nat. Rev. Mol. Cell Biol..

[100] Li Z, Berk M, McIntyre TM, Gores GJ, Feldstein AE (2008). The lysosomal-mitochondrial axis in free fatty acid-induced hepatic lipotoxicity. Hepatology.

[101] Gao C (2013). Phellinus linteus mushroom protects against tacrine-induced mitochondrial impairment and oxidative stress in HepG2 cells. Phytomedicine.

[102] Xu M (2014). Inhibition of cysteine cathepsin B and L activation in astrocytes contributes to neuroprotection against cerebral ischemia via blocking the tBid-mitochondrial apoptotic signaling pathway. Glia.

[103] Galluzzi L (2012). Molecular definitions of cell death subroutines: recommendations of the Nomenclature Committee on cell death 2012. Cell Death Differ..

[104] Degterev A (2005). Chemical inhibitor of nonapoptotic cell death with therapeutic potential for ischemic brain injury. Nat. Chem. Biol..

[105] Degterev A (2008). Identification of RIP1 kinase as a specific cellular target of necrostatins. Nat. Chem. Biol..

[106] Sun L (2012). Mixed lineage kinase domain-like protein mediates necrosis signaling downstream of RIP3 kinase. Cell.

[107] Gong Y (2019). The role of necroptosis in cancer biology and therapy. Mol. Cancer.

[108] Shi J, Gao W, Shao F (2017). Pyroptosis: gasdermin-mediated programmed necrotic cell death. Trends Biochem. Sci..

[109] He Y, Hara H, Nunez G (2016). Mechanism and regulation of NLRP3 inflammasome activation. Trends Biochem. Sci..

[110] Xie Y (2016). Ferroptosis: process and function. Cell Death Differ..

[111] Yu F (2016). The role of lysosome in cell death regulation. Tumour Biol..

[112] Stockwell BR (2017). Ferroptosis: a regulated cell death nexus linking metabolism, redox biology, and disease. Cell.

[113] Hou W (2016). Autophagy promotes ferroptosis by degradation of ferritin. Autophagy.

[114] Yang A (2014). Autophagy is critical for pancreatic tumor growth and progression in tumors with p53 alterations. Cancer Discov..

[115] Wang K, Tu Y, Wan JB, Chen M, He C (2019). Synergistic anti-breast cancer effect of pulsatilla saponin D and camptothecin through interrupting autophagic-lysosomal function and promoting p62-mediated ubiquitinated protein aggregation. Carcinogenesis.

[116] Bengsch F (2014). Cell type-dependent pathogenic functions of overexpressed human cathepsin B in murine breast cancer progression. Oncogene.

[117] Sevenich L (2011). Transgenic expression of human cathepsin B promotes progression and metastasis of polyoma-middle-T-induced breast cancer in mice. Oncogene.

[118] Withana NP (2012). Cathepsin B inhibition limits bone metastasis in breast cancer. Cancer Res..

[119] Zhao, Q., Gao, S. M. & Wang, M. C. Molecular mechanisms of lysosome and nucleus communication. *Trends Biochem. Sci*. 10.1016/j.tibs.2020.06.004 (2020). 10.1016/j.tibs.2020.06.004 PMC7572682 32624271

[120] Machado E (2015). Regulated lysosomal exocytosis mediates cancer progression. Sci. Adv..

[121] Lawrence RE, Zoncu R (2019). The lysosome as a cellular centre for signalling, metabolism and quality control. Nat. Cell Biol..

[122] Nixon RA (2013). The role of autophagy in neurodegenerative disease. Nat. Med..

[123] Stirnemann J (2017). A review of Gaucher disease pathophysiology, clinical presentation and treatments. Int. J. Mol. Sci..

[124] Boutry M, Morais S, Stevanin G (2019). Update on the genetics of spastic paraplegias. Curr. Neurol. Neurosci. Rep..

[125] Coffey EE, Beckel JM, Laties AM, Mitchell CH (2014). Lysosomal alkalization and dysfunction in human fibroblasts with the Alzheimer’s disease-linked presenilin 1 A246E mutation can be reversed with cAMP. Neuroscience.

[126] Lee JH (2015). Presenilin 1 maintains lysosomal Ca(2+) homeostasis via TRPML1 by regulating vATPase-mediated lysosome acidification. Cell Rep..

[127] Kinouchi K, Ichihara A, Itoh H (2011). Functional characterization of (pro)renin receptor in association with V-ATPase. Front. Biosci..

[128] Park JS, Blair NF, Sue CM (2015). The role of ATP13A2 in Parkinson’s disease: clinical phenotypes and molecular mechanisms. Mov. Disord..

[129] Funakoshi-Hirose I (2013). Distinct effects of methamphetamine on autophagy-lysosome and ubiquitin-proteasome systems in HL-1 cultured mouse atrial cardiomyocytes. Toxicology.

[130] Pal R (2016). NADPH oxidase promotes Parkinsonian phenotypes by impairing autophagic flux in an mTORC1-independent fashion in a cellular model of Parkinson’s disease. Sci. Rep..

[131] Weiss U (2008). Inflammation. Nature.

[132] Neurath MF (2019). Resolution of inflammation: from basic concepts to clinical application. Semin. Immunopathol..

[133] Oami T (2018). Blocking liver autophagy accelerates apoptosis and mitochondrial injury in hepatocytes and reduces time to mortality in a murine sepsis model. Shock.

[134] Ho J (2016). Autophagy in sepsis: degradation into exhaustion?. Autophagy.

[135] Ren C, Zhang H, Wu TT, Yao YM (2017). Autophagy: a potential therapeutic target for reversing sepsis-induced immunosuppression. Front. Immunol..

[136] Zhang H, Feng YW, Yao YM (2018). Potential therapy strategy: targeting mitochondrial dysfunction in sepsis. Mil. Med. Res..

[137] Yang X (2017). Hydroxytyrosol attenuates LPS-induced acute lung injury in mice by regulating autophagy and sirtuin expression. Curr. Mol. Med..

[138] De Meyer GR (2015). Autophagy in vascular disease. Circ. Res..

[139] Ma X, Godar RJ, Liu H, Diwan A (2012). Enhancing lysosome biogenesis attenuates BNIP3-induced cardiomyocyte death. Autophagy.

[140] Nagoor Meeran MF (2020). α-Bisabolol protects against β-adrenergic agonist-induced myocardial infarction in rats by attenuating inflammation, lysosomal dysfunction, NLRP3 inflammasome activation and modulating autophagic flux. Food Funct..

[141] Zhang S (2017). Knockout of Eva1a leads to rapid development of heart failure by impairing autophagy. Cell Death Dis..

[142] Ma X (2019). Transcription factor EB activation rescues advanced αB-crystallin mutation-induced cardiomyopathy by normalizing desmin localization. J. Am. Heart Assoc..

[143] Li J, Xiang X, Xu Z (2019). Cilostazol protects against myocardial ischemia and reperfusion injury by activating transcription factor EB (TFEB). Biotechnol. Appl Biochem..

[144] Dubland JA, Francis GA (2015). Lysosomal acid lipase: at the crossroads of normal and atherogenic cholesterol metabolism. Front. Cell Dev. Biol..

[145] Zhang H (2018). Lysosomal acid lipase and lipid metabolism: new mechanisms, new questions, and new therapies. Curr. Opin. Lipidol..

[146] Endo Y, Furuta A, Nishino I (2015). Danon disease: a phenotypic expression of LAMP-2 deficiency. Acta Neuropathol..

[147] Beertsen W (2008). Impaired phagosomal maturation in neutrophils leads to periodontitis in lysosomal-associated membrane protein-2 knockout mice. J. Immunol..

[148] Tanaka Y (2000). Accumulation of autophagic vacuoles and cardiomyopathy in LAMP-2-deficient mice. Nature.

